# How personal, behavioral, and environmental factors predict working in STEMM vs non-STEMM middle-skill careers

**DOI:** 10.1186/s40594-017-0079-y

**Published:** 2017-10-20

**Authors:** Matthew A. Cannady, Debra Moore, Elizabeth Votruba-Drzal, Eric Greenwald, Regie Stites, Christian D. Schunn

**Affiliations:** 1 Lawrence Hall of Science, University of California Berkeley, Berkeley, CA 94709 USA; 2 University of Pittsburgh, Pittsburgh, PA USA; 3 SRI International, Palo Alto, CA USA

**Keywords:** STEMM workforce, Middle-skill, STEMM career, STEMM education

## Abstract

**Background:**

Much of science, technology, engineering, mathematics, and medical (STEMM) education policy and research centers around developing the upper levels of the STEMM workforce sector. However, there are many positions in this workforce, “middle-skill careers,” that are largely responsible for executing the innovations and are largely ignored in STEMM education research.

**Results:**

Using data from the National Educational Longitudinal Study of 1988, we found differences in what predicts STEMM-related vs. non-STEMM careers across skill-level. For instance, underrepresented minorities and those exhibiting school transgressions are more likely to be working in middle-skill STEMM fields than in middle-skill non-STEMM fields as adults; the same is not true of the high-skill workforce.

**Conclusions:**

One-size-fits-all policies for broadening participation in the STEMM workforce across skill-level are unlikely to be successful. Further, programs that are designed to generate wonder and fascination with STEMM content may be successful in attracting more girls. However, to promote greater participation of individuals from traditionally underrepresented ethnic minority groups in STEMM, programs that support choices toward higher educational attainment, specifically four-year college degree attainment, are more likely to be successful.

## Introduction

Policy makers recognize that science-related careers are important, and mainlining these careers requires focused investments in education (Augustine 2005; Rothwell 2013). As the United States (US) President’s Council of Advisors on Science and Technology (PCAST) has stressed, “education will determine whether the United States will remain a leader among nations and whether we will be able to solve immense challenges in areas such as energy, health, environmental protection, and national security” (PCAST 2010, p. vii).

The focus of much of the policy and research in science-related education and the science-related workforce, in the US and around the world, centers around preparing individuals for the upper levels of the science-related workforce, meaning careers that require at least a four-year undergraduate degree in a science-related field. Many researchers even use science-related undergraduate degree attainment rather than job placements as the outcome measure of their studies (e.g., Maltese and Tai 2011; Eagan et al. 2010), assuming that it is a close proxy for entering the science-related workforce. This focus on the upper echelons addresses concerns about the underrepresentation of women (e.g., Ceci et al. 2014; Halpern et al. 2007) and people of color (e.g., Harper and Newman 2016) in science-related fields. The attention paid to the upper echelons of the science-related workforce is important because much of the innovation in these fields occurs at this level.

However, the exclusive focus on the upper echelons of the STEMM workforce, at the exclusion of other career levels across the science-related workforce ability distribution, is shortsighted because multiple sectors of the workforce drive innovation and productivity. In fact, only a small percentage of US students pursue advanced degrees in these disciplines and even fewer of these individuals go on to enter science-related careers (Cannady et al. 2014; Salzman et al. 2013). Beyond these upper echelons of the workforce, there are many individuals in the science-related workforce that are largely responsible for executing the innovations. These positions, defined as “middle-skill careers” and sometimes called “support occupations” (Solberg et al. 2012), require “some significant education and training beyond high school but less than a bachelor’s degree” (Holzer and Lerman, 2007, p. 8; Carnevale et al. 2013). The distinction between skill-levels of a career is defined by the typical education level of the individuals doing the work, rather than the sophistication of the work itself. Therefore, low-skill careers require little if any specific knowledge and therefore are generally filled by individuals with educations up to and including high school degrees. The boundary between middle-skill and low-skill careers is defined by the amount of education beyond high school, with middle-skill positions requiring on the job training, an associate’s degree, or equivalent. The boundary between middle-skill and high-skill careers is defined by a four-year college degree (Holzer and Lerman 2007, p. 8; Carnevale et al. 2013).

The size of the overall middle-skill workforce remains large (Holzer and Lerman 2009; Holzer 2015), despite the fact that the proportion of all careers that are middle-skill careers has been shrinking for the past three decades, from 59% in 1983 to 45% in 2012 (Tuzemen and Willis 2013). The exact size of the “middle-skill” workforce in science-related careers is difficult to determine, largely because it requires estimates of both the size of the middle-skill workforce and the size of the science-related career workforce. Estimates of the size of the middle-skill workforce in the United States range from 2% (Poole 2008) to 10% (Rothwell 2013) of the entire workforce in the United States. And the estimates of the size of the middle-skill workforce in science-related careers range from one-third (Poole 2008) to one-half (Rothwell 2013; Miller and Kimmel 2012) of science-related careers. Science-related middle-skill careers, many of which are in the high-tech industry, grew rapidly between 2003 and 2008 (Poole 2008), thus adding to the difficulty in estimating the size of current science-related middle-skill workforce and there is concern that the pool of individuals to fill further expansion of these positions is insufficient (Dennett and Modestino 2011).

Despite the large proportion of individuals working in middle-skill science-related careers and the importance of the work that they do, these careers and the individuals working in them are consistently excluded from most studies of the workforce in science-related careers (e.g., Tai, Liu, Maltese, & Fan, 2006; Cannady et al. 2014; Tyson et al. 2007). Therefore, little is known about this subgroup of the STEMM workforce, beyond economic and career-related studies estimating their size and contributions to the economy. In particular, there are few studies in the literature that consider the characteristics of individuals and their families related to selection into science-related middle-skills careers. Do the same knowledge, attitudinal, and demographic factors that predict participation in science-related middle-skill careers vs non-science-related middle-skill careers also predict participation in high-skill science-related careers vs non-science-related high-skill careers? As described below, there are many reasons to suspect that different factors will be important across these two skill-levels.

The goal of this study is to strengthen knowledge of the science-related middle-skill workforce. Many of the papers described above use variations in the definition of science-related careers and fields, but most often these include science, technology, engineering, and mathematics (STEM). We follow the approach of Miller and Solberg (2012) and many others (e.g., Lent et al. 2000; Eccles 1986; Maple and Stage 1991) to consider science-related fields to be those that are a part of the science, technology, engineering, mathematics and *medical* (STEMM) industries and often require educational attainment in these fields. We use data from the National Educational Longitudinal Study of 1988 (NELS:88), a dataset that has informed our understanding of high-skill STEMM careers (Maltese 2008). Our investigation will describe the demographic characteristics of the middle-skill STEMM workforce and high-skill STEMM workforce, and then compare these to the characteristics of the same skill level non-STEMM workforce. This analysis allows us to determine if the things that differentiate STEMM vs non-STEMM high-skill workers are the same as those that differentiate middle-skill STEMM and non-STEMM workers. This effort is important when considering whether educational policies focused on increasing the high-skill STEMM workforce are likely to also benefit the middle-skill workforce or whether differing policies will be needed for broadening participation in these two segments of the STEMM labor force.

## Theoretical framework

Recognizing that there are many factors influencing career choice, we draw upon the Social Cognitive Career Theory (SCCT) (Lent et al. 1994, 2000, 2002; Hartung et al. 2015), which has been used to investigate career interest development, selection of academic and career options, and persistence in educational and professional pursuits (e.g., Larson et al. 2015; Sax et al. 2016; Wang 2013). This framework, rooted in Bandura’s (1986) more general social cognitive theory, “emphasizes the means by which individuals exercise personal agency in the career development process, as well as extra-personal factors that enhance or constrain agency” (Lent et al. 1994, p. 79). Like other career development theories (e.g., Holland 1997; Dawis and Lofquist 1984; Osipow 1990), SCCT recognizes that a person’s agency in selecting and pursuing a career is nested within a societal context and that the person and society interact in determining an individual’s behavior. What differentiates SCCT from other career theories is the recognition that the career decisions are co-determined by the interaction of a person and their environment. That is, a person “influence[s] the situations that, in turn, affect their thoughts, affect and [subsequent] behavior” (Bandura 1986, p. 4 as quoted in Lent et al. 1994, p. 82).

Such a framework is useful in the current context as we consider the individual, environmental, and behavioral factors that relate to the decision to pursue a STEMM career over a non-STEMM career, while also attending to the individual, environmental, and behavioral influences on postsecondary academic attainment. In general, the environment (e.g., parental support), the individual (e.g., interests and skills), and their behaviors (e.g., completing college applications) each play a role in both STEMM vs. non-STEMM and bachelor’s degree vs. no bachelor’s degree decisions. However, the relative importance of each of these factors in shaping different kinds of STEMM career decisions is not well known.

## Literature

### Person: dispositions, attitudes, and characteristics matter

Individual expectations for future STEMM careers are important predictors of later STEMM outcomes. Robert Tai and colleagues (Tai, Liu, Maltese, & Fan, 2006; Tai, Ward, & Sadler, 2006) found that eighth-grade science or engineering career expectations were positively related to STEMM degree completion. Moreover, the importance of interest and identity seems to increase as one progresses along the pathway toward a STEMM career. For example, several studies have reported that students with high levels of preparation and skill in math and science select out of STEMM majors or may not choose STEMM careers unless they are sufficiently interested in the discipline (Besterfield-Sacre et al. 1997; Dick and Rallis 1991; Lubinski and Benbow 2006; Masnick et al. 2009). Consistent with these findings, research drawing on the Study of Mathematically Precocious Youth (e.g., Lubinski et al. 2006) has reported that interest and identity play critical roles for STEMM degree earners who go on to careers in the highest echelon of STEMM professions. This suggests that intrinsic interest in the discipline may be a particularly important differentiation among high-performing individuals.

Further, research has shown substantial differences between genders in the pursuit of STEMM degrees (Nicholls et al. 2010) and advancement in the STEMM academic career path (Rapoport et al. 2004). Some researchers attribute this difference to the reconciliation of multiple identities, a complex undertaking that white males in the academy grapple with less often than their non-white and female counterparts (Carlone and Johnson 2007; Gainor and Lent 1998; Zirkel 2002). Others have argued that women, when matched on mathematics ability, tend to have higher verbal ability than men, which creates more non-STEMM career opportunities for women (Wang et al. 2013). However, when including medical fields in the outcome, STEMM, the gender differences are largely muted (Kimmel et al. 2012).

### Behaviors: competencies and math and science course-taking matter

Math and science competence are good statistical predictors of some STEMM pathway outcomes. Focusing on mathematics skill, several studies have drawn on high school SAT-Math scores in nationally representative datasets (e.g., Astin and Astin 1992); Nicholls et al. 2010) and show that math ability predicts academic achievement in high school, college, and throughout science-related graduate degrees (Achter et al. 1999; Benbow et al. 2000; Lubinski and Benbow 2006; Lubinski et al. 2006). Further, math performance is predictive across STEMM subfields (Donovan and Wheland 2009) and for important demographic subgroups (Bonous-Hammarth 2000; Sondgeroth and Stough 1992). For example, Wai et al. (2005) demonstrated that SAT-Math scores predict later science-related career selection and productivity among the highest echelon of professionals, using tenure, publications, and patents as proxies for productivity.

In addition to math ability, perceptions of math competence are correlated to key outcomes, including high school course taking, college major selection, and STEMM degree persistence (Leslie et al. 1998; Mau 2003; Nicholls et al. 2010). Math confidence seems to be particularly important for women, who often report lower math self-concept than their male peers despite having equivalent or higher achievement (Leslie et al., 1998). While general academic self-concept has also been associated with desirable STEMM outcomes, findings suggest that it is a less powerful and less robust indicator than math self-concept (Astin and Astin 1992; Mau 2003). While personal measures of math ability and interest are powerful predictors of success in STEMM education and careers, so too are course-taking choices and performance. As a first step, students who pass algebra by eigth grade are more likely to select and succeed in high-level math courses in high school (Adelman 1999, 2006). Then, students who select and successfully complete high-level math courses in high school tend to perform better in college STEMM courses and are more likely to earn (bachelors and higher) STEMM degrees (Astin and Astin 1992; Leslie et al. 1998; Nicholls et al. 2007; Tai et al. 2005; Tai, Liu, Maltese, & Fan, 2006; Wai et al. 2005).

Several large-scale studies have reported that science course-taking patterns are associated with key STEMM outcomes as well (e.g., Adelman 1999, 2006; Tyson et al., 2007). For instance, Farmer and colleagues (1995) found that passing an elective high school science course predicts college performance and persistence in STEMM undergraduate majors. However, other large-scale studies suggest that the predictive power of science course taking in high school may be less generalizable across STEMM subfields than is math course taking. Studies by Sadler and Tai (2001, 2007) reported that advanced course taking in high school physics specifically predicts college physics performance and degree persistence. A separate study (Tai et al. 2005) found a similar pattern for chemistry. Neither study found that the additional high school course work was a significant predictor of success in STEMM subfields outside the corresponding domain. For example, students who take an extra year of high school physics were not more likely to persist in earning bachelor degrees in biology.

Taken together, these studies suggest a fairly common pathway toward STEMM degrees: take algebra by grade 8, take calculus and at least one advanced science course in high school, proceed straight to college after high school, select a STEMM major, and graduate with that major in 4 to 5 years. However, these studies focus on the upper end of the STEMM workforce, that is, those working in STEMM fields who have earned at least a bachelor’s degree. They do not consider links between these skills and course-taking patterns and careers at the middle level of the STEMM workforce. It may be that for associates degrees, which are less selective (i.e., accept applicants with weaker academic preparation), advanced mathematics and science coursework will be less critical. However, if such experiences and performance drive attitudes toward STEMM, they may in fact remain important experiences.

### Environment: context matters

Socioeconomic, cultural, and experiential factors also play a role in students’ pursuit of, persistence in, and, ultimately, attainment of STEMM degrees. For instance, studies have concluded that knowledge of the education system (course requirements, college application process, how to finance one’s education, pre-requisites for certain majors, and an overall knowledge of how to navigate the ‘system’) is just as important as academic aptitude for not only gaining access to postsecondary education but also successfully completing a degree (Castleman and Page 2014; Brody 2006; Eagan et al. 2010). These problems are especially pertinent for low socioeconomic students who have less access to advanced math and science courses in high school and their families are less able to invest resources into their education (Oakes 1990).

### Focus on STEMM

Defining science-related careers is not simple, with the largest debate centering around the inclusion or exclusion of careers in the medical or health fields. Within the United States, the National Science Foundation (NSF) has formally chosen to consider science-related fields as those fields pertaining to science, technology, engineering, and mathematics, thereby creating the ubiquitous acronym STEM. Given that many educational researchers in the US seek funding from the NSF, much of the research they produce excludes medical and health fields from their focus of study. Part of the argument for this exclusion is the belief that individuals in the medical or health fields rarely contribute to research or the creation of new knowledge, which is seen as a defining feature of the field of science. However, using that criteria for inclusion as science-related careers should exclude many if not most engineers. Further, excluding medical and health fields from science-related careers excludes many of the actual careers youth aspire to enter and that the exclusion of medical and health careers is largely gendered both in the perspective of those careers as being “motherly” and that there is far greater gender balance in these fields than, for example, in engineering (Kimmel et al. 2012). For this analysis, we have chosen to append science, technology, engineering, and mathematics fields to include the medical field and note this addition by using the acronym STEMM.

### Focus on middle-skill careers

Middle-skill careers are widely diverse, but generally require some amount of postsecondary education or training. This includes associate’s degrees, vocational certificates, significant on-the-job training, previous work experience, or generally “some college” less than a bachelor’s degree (Holzer and Lerman 2007). Common careers used as examples of STEMM middle-skill careers include radiology technicians, engineering technicians, and electricians.

Little is known about the status of the labor market in the middle-skill positions, most especially the STEMM middle-skill workforce. It is very difficult to estimate the current size of the STEMM middle-skill workforce in the US. The overall STEMM workforce is estimated to be 6% (Landivar 2013) to 20% (Rothwell 2013) of the entire US workforce, and middle-skill careers are estimated to account for just under half of all occupations in the US (Holzer and Lerman 2009; Tüzemen and Willis 2013). However, there is no consensus on the definition of middle-skill careers, either in the overall workforce or within the STEMM fields. Further, there are also varying definitions of what counts as a STEMM career. As a result, estimates of what proportion of all STEMM careers are low, middle, or high skill vary. This lack of clarity on the size and composition of the middle-skill STEMM workforce has several explanations. First, the Bureau of Labor Statistics does not publish estimates of job openings by skill category (Kochan et al. 2012; Holzer and Lerman 2007). Second, middle-skill careers have not been the primary focus of STEMM education initiatives and therefore have largely been ignored by researchers. Into this void of clarity, Holzer and Lerman (2007) offered a definition of all jobs within each of the ten broad clusters of occupational categories as high-skill (managerial and professional/technical occupations), middle-skill (clerical, sales, construction, installation/repair, production, and transportation/material moving occupations), or low-skill (service and agricultural occupations) and recognized that these categories largely aligned with educational levels. Since then, several researchers have followed their classifications and definitions of these careers (McDaniel and Kuehn 2013; Solberg et al. 2012). For instance, Kochan et al. (2012) used Holzer and Lerman’s definition of career skill levels and estimated that middle-skill careers will account for 47% of all new job openings in the US from 2010 to 2020 (Kochan et al. 2012). In an attempt to update that research, we used the same methods and estimated that 42% of all job openings in the US between 2014 and 2024 will be in the in the clusters Holzer and Lerman identify as middle-skill. Further, throughout this study, we use Holzer and Lerman’s (2007) definition of middle-skill careers due to an increased use in the literature and clear guidance for use in the relevant data sets.

In an attempt to respond to the need for greater clarity and better information regarding an important component of the US STEMM workforce, we sought answers to the following research questions:

1. What are the characteristics of the overall STEMM workforce that differentiate them from the non-STEMM workforce?
2. Are the STEMM vs. non-STEMM differentiating characteristics the same for high-skill workers as for middle-skill workers?

Some of the past studies of predictors of STEMM workforce have a confounded level (i.e., requiring a bachelor’s or higher degree vs. requiring vocational training or an associate’s degree) and domain (STEMM or other). For example, advanced high school coursework in difficult topics may generally predict applying for and completing bachelor degrees rather than specifically STEMM degree completion. This study is interested in exploring the STEMM vs non-STEMM decisions for each of the middle-skill and high-skill career levels. The study is not interested in determining the difference between middle-skill and high-skill STEMM workers, as the difference between these two is defined by their four-year degree status and is far more likely to be determined by factors with four-year degree attainment than anything specific to STEMM.

## Methods

### Data

This study draws data from the National Educational Longitudinal Study of the Eighth-Grade Class of 1988 (NELS:88), which follows a nationally representative sample of eighth graders for 12 years, through their postsecondary education and into their careers (National Center for Education Statistics, 1988, 1990, 1992, 1994, and 2000). The data set has been used extensively for longitudinal studies aimed at informing STEMM education policies (e.g., Plunk et al. 2014) and was used to conduct many of the studies cited earlier in the literature review section. Importantly, for the purposes of this study, the NELS:88 collects information on early career interest, high school course taking, plans for secondary school enrollment, college completion, including major(s), and subsequent employment.

The NELS:88 used a two-stage national probability sample of approximately 24,600 eighth graders enrolled in public and private schools in 1988. A subset of these students were resurveyed in 1990 (10th grade), 1992 (12th grade), 1994 (19–20 years old), and again in 2000 (25–26 years old) when many had entered the workforce. Altogether 12,145 individuals completed the fourth follow-up survey in 2000 (Curtin et al. 2002).

Our sample consists of individuals who were a part of the eighth-grade cohort of 1988, had high school transcript data, and completed some postsecondary education. Our operational definitions for groups used in the analysis along with missing responses to variables used in the analysis reduced the overall unweighted sample size to roughly 3860 (weighted sample sizes are rounded to the nearest 10). This represents 32% of the total pool with data from the fourth follow-up survey. The chief limiting variable was the restriction that participants complete some postsecondary education. Incomplete demographic variables caused most of the additional loss. Analyses of career outcomes in longitudinal studies are typically restricted in this way.

### Outcome variables

Of primary interest in this study were career categories generated from the interviews conducted at age 26. The dataset contains some career codes that have been used in past research; however, more refined codes were needed to differentiate careers into: STEMM middle-skill career, STEMM professional, non-STEMM middle-skill career, and non-STEMM professional. In order to classify participants, definitions of each category were created. First, a list of definitions and examples of STEMM middle-skill careers was compiled based on information from the Occupational Information Network (O*NET) Resource Center (Table 1), which is an online database with occupational definitions developed by North Carolina Employment Security Commission under a grant from the US Department of Labor/Employment and Training Administration. Using the occupation codes already in NELS:88, we filtered 3875 respondents by occupation codes determined to be STEMM-related (Table 2) and had at least some postsecondary education. This resulted in a list of participants who could potentially be working in a STEMM career, either as the high-skill or middle-skill levels. Two researchers then manually read through each of the job titles for these respondents and coded them as working in STEMM or not in STEMM and as requiring middle-skill or as high-skill.

**Table 1:** STEMM middle-skill career definitions

Engineering technician	Life science technician	Physical science technician	Medical and clinical health technician	Computer/mathematical technician	Laboratory technician
Occupations that entail assisting on engineering work but do not typically include engineering design or high-level analytical work. Many of these occupations are open to applicants with associates degree and/or appropriate professional certification.	Occupations that entail assisting in life science work or research but do not typically include design or high-level analytical work. Many of these occupations are open to applicants with associates degree and/or appropriate professional certification.	Occupations that entail assisting in physical science work or research but do not typically include design or high-level analytical work. Many of these occupations are open to applicants with associates degree and/or appropriate professional certification.	Occupations that entail assisting in healthcare diagnosis and treatment, including operation of advanced medical equipment, and may or may not include direct provision of care to patients. Level of education and certification required varies.	Occupations that entail development, installation, troubleshooting, and/or maintenance of computer/IT hardware or software. Many of these occupations are open to applicants with associates degree and/or appropriate professional certification.	Occupations that entail lab work but are not clearly specified as life science, physical science, or biomedical/clinical laboratory work. In most cases, should be accessible to someone with an Associate’s degree or professional certification.

**Table 2:** STEMM occupation codes in NELS:88

Medical practice professionals	
Medical licensed professionals	
Medical services	
Human services professionals	
Engineers architects software engineers	
Scientist, statistician professionals	
Research assistants/lab technicians	
Technical/professional workers, other	
Computer systems/related professionals	
Computer programmers	

A STEMM high-skill worker was further defined as someone working in the STEMM field according to their job category in NELS:88 (Table 2) and who held a bachelor’s degree in a STEMM field (Table 3). Any participant manually coded as having a STEMM middle-skill career and was subsequently determined to have a bachelor’s degree was removed from the middle-skill careers category and placed in the STEMM professional/high-skill category to be consistent with the definitional category of high-skill careers.

**Table 3:** STEMM major codes in NELS:88

Agriculture	Health-medicine
Agricultural science	Health-veterinary medicine
Natural resources	Nursing-registered nurse
Forestry	Health-health/hospital Administration
Computer programming	Health-public health
Computer and information sciences	Health-preparatory programs
Engineering-electrical	Health-dietetics
Engineering-chemical	Health-pharmacy
Engineering-civil	Health-optometry
Engineering-mechanical	Biological science-zoology
Engineering-all other	Biological science-botany
Engineering technology	Biological science-biochemistry
Health/allied-dental/medical technology	Biological science-all other
Health/allied-Therapy and mental health	Mathematics-statistics
Health/physical education/recreation	Mathematics-not statistics
Nursing-nurse assisting	Interdisciplinary-integrated science
Agriculture	Interdisciplinary-all other
Agricultural science	Physical sciences-chemistry
Health/allied-general and other	Physical sciences-earth science
Nursing-nursing, post-RN	Physical sciences-physics
Health-audiology	Physical sciences-not Chemistry/physics/earth
Health-clinical health science	Transportation-air
Health-dentistry	Transportation-not air

Participants who had earned a college degree, whether it was STEMM or non-STEMM, but were working in a field with a job code not identified as STEMM were coded as a non-STEMM professional. Non-STEMM middle-skill careers were defined as individuals with some postsecondary education but no bachelor’s degree and were not working in a STEMM middle-skill career.

### Predictors and control variables

#### Academic behavioral variables

Several behavioral characteristics were included in our analysis to capture behaviors that are specific to school and science. We made this decision assuming that an individual’s science achievement and course selection manifest from the behaviors individuals exhibit toward science (Updegraff et al. 1996). Science achievement was measured using the science item response theory (IRT) theta scores derived from a science assessment that was developed by the Educational Testing Service. A broader measure of academic achievement was used that had combined IRT theta scores of assessments of math and reading skills. Both of these achievement scores came from the initial wave of data collection (eighth grade, Rock and Pollack, 1991). Two variables were created to indicate whether a student had completed an advanced math and science classes. Completion of an advanced math class was defined as having taken and passed either calculus or pre-calculus. Completion of an advanced science class was defined as having taken and passed biology, chemistry, and physics at regular or advanced placement (AP) levels; in the US, only two sciences courses are required for graduation in most high schools.

#### Personal variables

A variety of personal characteristics that have been previously linked to career selection were included: four science-related attitudinal variables, two categories of student dispositional variables, and several student demographic variables. Attitude toward math class was an average of two items from the NELS:88 survey (I look forward to math class; I think math will be important in my future). Similarly, attitude toward science class was calculated by taking an average of the analogous two items relating to science class. Additionally, whether or not the student wanted to be a scientist or engineer when they were adults, as well as an indicator variable as to whether or not the student expected to graduate from college, were included as predictors.

Additional general academic dispositional variables that regularly predict academic success were included (Table 4). Two of these scales were created within the NELS:88 base year survey self-concept and locus of control (Rock and Pollack 1991). Student self-concept (Marsh 1994) was a factor score of the nine-item scale used by NELS:88 researchers to measure self-concept and had a high internal reliability of (*α* = 0.98). Items in this scale included “I feel good about myself”; “I feel I am a person of worth, the equal of other people”; “I feel I do not have much to be proud of” (reverse coded). Likewise, student locus of control (Mau 2003) was measured using a factor score that combined responses to seven items into the NELS:88 scale score. It also had a high internal reliability (*α* = 0.98). This scale included items such as “I don’t have enough control over the direction my life is taking”; “In my life, good luck is more important than hard work” (reverse coded); “When I make plans, I am almost certain I can make them work.” In addition to the scales within the NELS:88 data, we created a measure of school transgressions that is highly reliable (*α* = 0.99) (Parker and Benson 2004). This scale included items such as “sent to the office with school work problems”; “parents received warning about behavior”; “got into a fight with another student.”

**Table 4:** NELS:88 survey items used to create scale scores

Items used for self-concept score (α = .98)	I feel good about myself
	I’m a person of worth, equal of others
	I am able to do things as well as others
	On the whole, I am satisfied with myself
	I certainly feel useless at times
	At times I think I am no good at all
	I feel I do not have much to be proud of
	I feel good about myself
	I’m a person of worth, equal of others
Items used for locus of control score (*α* = .98)	I don’t have enough control over my life
	Good luck more important than hard work
	Every time I get ahead something stops me
	Plans hardly work out, makes me unhappy
	When I make plans I can make them work
	Chance and luck important in my life
	I don’t have enough control over my life
Items used for externalizing problems score^a^ (*α* = .99)	Student sent to office for misbehaving
	Student sent to office with school work problems
	Parents received warning about attendance
	Parents received warning about grades
	Parents received warning about behavior
	Student got into fight with another student

^a^Items were recoded before creating the factor score

Three student demographic variables commonly associated with underrepresentation in STEMM were included in the analysis, gender, native language, and race/ethnicity. All were taken from the eighth grade interview. Gender was coded dichotomously, consistent with how the data was collected in 1988. Students’ native language was represented with a binary variable that indicated whether their primary language is English versus another non-English language. Student race/ethnicity was coded with a binary variable indicating whether students are a member of an overrepresented STEMM race/ethnic group (i.e., White or Asian) or an underrepresented STEMM race/ethnic group (e.g., Black, Hispanic, Native American).

#### Environmental variables

In an attempt to capture the environment individuals experienced while maturing into their postsecondary and career decisions, we included variables that captured important household characteristics. These included parental educational attainment, nativity status, marital status, and economic status, as well as, parental expectation that the student would graduate from college. All five of these variables were coded using data from eighth grade. More specifically, parental educational attainment was measured using an indicator or whether at least one parent earned a college degree. Parental nativity status was coded dichotomously with an indicator of whether both parents were born in the United States. Marital status was also coded with a binary variable reflecting whether the student’s parents were married in eighth grade. Family economic status was measured with an indicator of whether the household income fell below the Federal Poverty Line. Finally, parental educational expectations were assessed with a single indicator of whether at least one parent expected the student to graduate from college.

Descriptive statistics of all predictor and control variables are shown in Table 5.

**Table 5:** Descriptive statistics for STEMM high and middle-skill careers and non-STEMM high and middle-skill careers

Variable categories	Predictor and control variables	Variable status	STEMM high-skill	STEMM middle-skill	Non-STEMM high-skill	Non-STEMM middle-skill
Unweighted sample size^a^			750	170	3140	1130
Family variables	Parents married	Yes	94%	86%	89%	82%
		No	6%	14%	11%	18%
	Parent has degree	Yes	55%	28%	54%	25%
		No	45%	72%	46%	75%
	Parents US born	Yes	83%	83%	89%	91%
		No	17%	17%	11%	9%
	Low income	Yes	11%	29%	14%	28%
		No	89%	71%	86%	72%
	Parent expects college graduation	Yes	89%	72%	93%	75%
		No	11%	28%	7%	25%
Student demographics	Gender	Male	56%	80%	43%	61%
		Female	44%	20%	57%	39%
	Native language	English	92%	90%	95%	94%
		Non-English	8%	10%	5%	6%
	Underrepresented minority	Yes	10%	24%	9%	15%
		No	90%	76%	91%	85%
Student academic characteristics	Took advanced math	Yes	19%	9%	10%	2%
		No	81%	91%	90%	98%
	Took advanced science	Yes	58%	24%	40%	16%
		No	42%	76%	60%	84%
	Wants to be scientist or engineer	Yes	17%	10%	8%	5%
		No	83%	90%	92%	95%
	Attitude toward math	(s.e.)	.140 (.030)	.091 (.060)	.017 (.016)	−.032 (.025)
	Attitude toward science	(s.e.)	.163 (.034)	.099 (.074)	.002 (.017)	−.013 (.029)
Student achievement	Composite score	(s.e.)	58.97 (.530)	52.16 (1.09)	57.20 (.318)	48.78 (.654)
	Science IRT	(s.e.)	50.89 (.640)	48.38 (.937)	49.31 (.243)	43.85 (.618)
Student dispositional characteristics	Self-concept	(s.e.)	.149 (.020)	.149 (.063)	.157 (.015)	0.01 (.029)
	Locus of control	(s.e.)	.178 (.025)	.232 (.076)	.144 (.021)	0.03 (.037)
	Good school behaviors	(s.e.)	.135 (.027)	− .222 (.059)	.142 (.017)	− 0.27 (.045)
	Expects to graduate college	Yes	90%	71%	92%	67%
		No	10%	29%	8%	33%

^a^Rounded to the nearest 10 to protect anonymity

### Analysis

A two-fold analysis was undertaken to (1) differentiate STEMM vs. non-STEMM characteristics overall and then (2) differentiate for those working as a high-skilled STEMM professional from high-skilled non-STEMM professionals and those working in a STEMM middle-skill career compared to those working in a non-STEMM middle-skill career.

The selected personal, environmental, and behavioral measures were first entered into a logistic regression analysis to determine how well they could differentiate between individuals working in STEMM fields and non-STEMM fields overall. Collinearity diagnostics showed no issues among the predictor and control variables. All logistic regression models were conducted using the F4PNLWT sampling weight and the complex sampling design options (svy commands) within STATA 12. This sampling weight is for the fourth follow-up complete panel weight for respondents at all five NELS:88 data collection points. Using these sampling weights makes the findings of this study generalizable to a nationally representative sample of eighth graders in the spring of 1988.

Next, these same indicators were used in a multinomial regression with a four-category outcome (STEMM professional, non-STEMM professional, STEMM middle-skill worker, non-STEMM middle-skill worker) to illuminate STEMM vs. non-STEMM differences separately for high-skill professions and middle-skill careers. Pairwise comparisons were used to determine which of these variables were associated with differences between STEMM vs. non-STEMM middle-skill workers and STEMM vs. non-STEMM high-skill workers. The multinomial regression model, with non-STEMM middle-skills careers serving as the base outcome, was again conducted using the F4PNLWT sampling weight and the complex sampling design options (svy commands) within STATA 12. The “listcoeff” command was used to perform all relevant comparisons between the four groups in the model.

The variance inflation factor (VIF) was examined for each of the predictor variables, as a test of multicollinearity within the model. No variables had a VIF greater than 2.5 and were therefore deemed to be sufficiently independent contributions to the model.

## Results

### Logistic regression: what characteristics differentiate the STEMM workforce from the non-STEMM workforce?

The overall prediction of STEMM-related career (whether high- or middle-skill) by the predictor variables was statistically significant; *F* (19, 3814) = 8.53, *p* < 0.001. At least one variable from each of the three aspects of social cognitive career theory was significantly predictive of who was working in a STEMM career: married parents as an environmental variable; gender, self-concept, and expecting to be a scientist or an engineer as person variables, and advanced courses in math and science, and higher Science IRT theta scores as behavioral variables (Table 6).

**Table 6:** Summary of logistic regression analysis for variables predicting working in a STEMM career vs working in a non-STEMM career

STEMM career vs. non-STEMM career	B	Std. Err.	e^B
Behavioral variables			
Comp. math and reading IRT theta	0.00	0.01	1.00
Science IRT theta	0.04***	0.01	1.04
Advanced math course	0.48**	0.16	1.61
Advanced science course	0.41**	0.12	1.51
Personal variables			
Attitude toward math	0.32	0.30	1.37
Attitude toward science	0.00	0.23	1.00
Science or engineer career expectation	0.37*	0.16	1.45
College graduation expectation	− 0.12	0.17	0.89
Self-concept	− 0.57*	0.26	0.57
Locus of control	0.25	0.19	1.28
Negative/risky behaviors	0.12	0.12	1.13
Female	− 0.43***	0.11	0.65
English is Native Language	0.05	0.28	1.05
Underrepresented minority	0.13	0.19	1.14
Environmental variables			
Parent education	0.03	0.11	1.03
Parent Native US citizen	− 0.35	0.20	0.70
Parents married	0.46*	0.19	1.58
College graduation expectation of parent	− 0.22	0.18	0.80
Low income	− 0.15	0.16	0.86
Constant	− 3.23***	0.54	0.04

Binary coded variables are 1 for yes and 0 for no

*Note: e* ^*B*^ exponentiated *B*

*p < .05; **p < .01; ***p < .001

In contrast, there was no significant additional prediction of pursuing a STEMM-related career by parental education level, being an underrepresented minority, having native-born parents, low income status, student native language, student or parental expectation of college graduation, general achievement, attitude toward math or science class, student self-concept, or engaging in poor academic behaviors. Note that many of these variables in isolation are associated with pursing a STEMM career on their own, but these associations disappeared once other variables were included as controls. For example, attitude toward math or science class is different as a mean between STEMM and non-STEMM groups, but does not add significantly as a predictor when other controls are included.

### Multinomial regression

Are the STEMM vs. non-STEMM differentiating characteristics the same for high-skill workers as for non-middle-skill workers*?*

There was an overall significant prediction of group membership: STEMM professional, non-STEMM professional, working in a STEMM middle-skill career, or working in a non-STEMM middle-skill career; *F* (57, 3776) = 10.38, *p* < .001. All possible pairwise comparisons revealed significant differences in prediction of variables by group. However, low-income status, student native language, parental nativity status, attitude toward math class, and student locus of control were not significant predictors.

First, we describe the difference between STEMM middle-skill and STEMM high-skill workers. Those working in high-skill STEMM positions are more likely to be female, have married parents, have a parent with a bachelor’s degree, exhibit school conforming behaviors, expect to work as a scientist or engineer, score higher on science achievement tests, and take advanced math and science courses than STEMM middle-skill workers.

Second, comparing STEMM middle-skill workers to non-STEMM middle-skill workers found that STEMM middle-skill workers were more likely to have higher science IRT thetas, taken an advanced math course, more likely to have engaged in poor academic behaviors, be male, and be from a traditionally underrepresented minority in STEMM (Table 7 shows the significant variables from post-hoc analyses of the multinomial regression, non-significant variables, married parents, parental education level, parental nativity status, low-income status, parent’s college graduation expectation, student native language, advance science course taking, attitude toward science or math class, expecting to be a scientist or engineer, composite math and reading score, self-concept, locus of control, and if they expect to graduate from college are omitted from the post-hoc tests and the table). It is important to note the direction of the ethnicity effects: traditionally underrepresented minorities are actually more likely to be in STEMM middle-skill careers than in non-STEMM middle-skill careers.

**Table 7:** STEMM middle-skill careers vs. non-STEMM middle-skill careers

	B	Std. Err.	e^B
Behavioral variables			
Comp. math and reading IRT theta	0.01	0.02	0.50
Science IRT theta	0.05*	0.02	0.51
Advanced math course	1.07†	0.56	0.74
Advanced science course	− 0.05	0.35	0.49
Personal variables			
Attitude toward math	− 0.06	0.72	0.48
Attitude toward science	− 0.10	0.56	0.47
Science or engineer career expectation	0.42	0.42	0.60
College graduation expectation	0.12	0.31	0.53
Self-concept	− 0.87	0.56	0.30
Locus of control	0.66	0.44	0.66
Negative/risky behaviors	0.42†	0.25	0.60
Female	− 0.94**	0.32	0.28
English is Native language	0.61	0.70	0.65
Underrepresented minority	0.72*	0.35	0.67
Environmental variables			
Parent education	− 0.02	0.31	0.49
Parent Native US citizen	− 0.45	0.42	0.39
Parents married	0.06	0.38	0.51
College graduation expectation of parent	− 0.23	0.32	0.44
Low income	0.11	0.32	0.53
Constant	− 4.72	1.27	0.01

Binary coded variables are 1 for yes and 0 for no

*Note: e* ^*B*^ exponentiated *B*

†*p* < 0.1; *p < .05; **p < .01;

Comparing STEMM professionals to non-STEMM professionals (see Table 8) found that STEMM professionals were more likely to be male, have married parents, have higher science IRT thetas and enroll in advanced math and science courses and were expected to be a scientist or engineer than non-STEMM professionals. Non-STEMM professionals, however, had higher self-concept scores and were more likely to expect to graduate from a college than STEMM professionals. Thus, only general science knowledge predicted STEMM careers for both high-skilled and middle-skilled careers.

**Table 8:** Non-STEMM high-skill careers vs. STEMM high-skill careers

	B	Std. Err.	e^B
Behavioral variables			
Comp. math and reading IRT theta	− 0.01	0.01	0.50
Science IRT theta	0.03**	0.01	0.51
Advanced math course	0.45**	0.17	0.61
Advanced science course	0.45**	0.14	0.61
Personal variables			
Attitude toward math	0.25	0.35	0.56
Attitude toward science	0.21	0.26	0.55
Science or engineer career expectation	0.46*	0.18	0.61
College graduation expectation	− 0.52*	0.20	0.37
Self-concept	− 0.68*	0.30	0.34
Locus of control	0.31	0.22	0.58
Negative/risky behaviors	0.09	0.15	0.52
Female	− 0.25*	0.13	0.44
English is Native language	0.11	0.33	0.53
Underrepresented minority	0.04	0.23	0.51
Environmental variables			
Parent education	− 0.10	0.13	0.48
Parent Native US citizen	− 0.37	0.25	0.41
Parents married	0.74**	0.24	0.68
College graduation expectation of parent	− 0.32	0.21	0.42
Low income	− 0.21	0.18	0.45
Constant	− 2.54***	0.64	0.07

Binary coded variables are 1 for yes and 0 for no

*Note: e* ^*B*^ exponentiated *B*

†*p* < 0.1; *p < .05; **p < .01; ***p < .001

## Conclusion

Middle-skill careers require education and training beyond high school but less than a bachelor’s degree, and individuals working in these positions are crucial for executing technological innovation. Middle-skill careers have largely been excluded from science education research, which has tended to focus on broadening participation in the high-skill STEMM workforce to women and traditionally underrepresented minority populations. In this study, we looked for differences in the compositions of the STEMM and non-STEMM workforce across the high-skill and middle-skill levels.

### Consistency across skill level for STEMM career entry

In this study, we examined factors that predict STEMM career entry at different skill levels in several ways. Across all levels of STEMM careers, we found that general science literacy predicts entry into a STEMM career. That is, having higher science knowledge predicts STEMM careers for both high-skill and middle-skill careers. Thus, efforts to enhance science literacy are promising for increasing the number of STEMM workers across skill levels. Similarly, advanced course taking in mathematics, the only variable in the analysis specific to math achievement, increased the propensity that an individual worked in STEMM careers at all skill levels. This suggests that mathematics aptitude is also important for promoting careers in STEMM; thus, supporting math achievement also remains important to support STEMM career entry.

### Differences across skill level in STEMM career entry

Several factors emerged that were unique in predicting STEMM career entrance within each skill level. We found eight such factors within the high-skill workforce and four within the middle-skill workforce. Looking across these 12 factors, it is clear that a single strategy to broaden the STEMM workforce across skill levels is unlikely to work and instead our efforts must be multifaceted.

For instance, like others, we found that among the high-skill workforce, advanced science courses (Tyson et al. 2007) and STEMM career expectation (Tai, Liu, Maltese, & Fan, 2006) distinguished future STEMM workers from non-STEMM workers. Further, variables related to college graduation expectations and self-concept were predictive of who would enter a STEMM career. We also found family variables, such as having married parents and native-born parents, were useful predictors of STEMM career entry. However, none of those variables were useful in differentiating STEMM vs non-STEMM workers when it came to the middle-skill workforce. Instead, underrepresented minorities and those more frequently exhibiting school transgressions while in eighth grade were more likely to be working in middle-skill STEMM fields than in middle-skill non-STEMM fields as adults.

So, while interventions focused on promoting advanced science course taking or STEMM career exploration might be useful for predicting entry into high-skilled STEMM careers, this is not true for middle-skill careers. Given that individuals with more frequent school transgressions while in eighth grade were more likely to enter STEMM middle-skill careers, perhaps within the middle-skill population, STEMM represents an opportunity for exploration and an alternative to more traditional pathways through schooling. Science may present itself as an area to feel successful as those working in STEMM middle-skill careers have higher science achievement scores than their peers working in non-STEMM middle-skill careers. Out-of-school learning, in informal environments, may play a larger role in predicting STEMM career entry for individuals at the middle-skill level than it does for those who work in high-skill careers and informal experiences are rarely, if ever, considered critical factors, worthy of educational policy, in preparation of the future STEMM workforce.

### Traditionally underrepresented populations in STEMM

We also found an interesting pattern in the distribution of individuals from traditionally underrepresented minority populations in STEMM. Some ethnic minority groups that are traditionally underrepresented in the high-skill STEMM workforce relative to the population as a whole are actually overrepresented in the middle-skill STEMM workforce relative to the middle-skill non-STEMM workforce. In the United States, racial/ethnic minority populations are commonly considered to be underrepresented within STEMM education and STEMM careers. In other words, there are lower proportions of individuals from these racial/ethnic groups than there are in the population as a whole. In our study, when focusing on just high-skill careers, we did not find a difference in the proportion of underrepresented minority populations between the STEMM and non-STEMM workforce. This implies that the underrepresentation of particular minority populations is a four-year college degree attainment issue, rather than an issue specific to STEMM fields. The underrepresentation of racial/ethnic minority populations in high-skill STEMM careers is similar to the average underrepresentation of the same minority populations in high-skill non-STEMM careers, such as law, banking, marketing, and journalism.

Although gaps in educational attainment have been narrowing significantly in recent years, Black and Hispanic students are still less like to enter postsecondary education within 8 years of expected high school graduation. Among high school sophomores in 2002, rates of entry into postsecondary education were 79% for Hispanic youth, 82% for Black youth and, 87% for White youth (Cahalan et al. 2016). In recent years, significant progress has been made in narrowing gaps in degree completion by race/ethnic status, there are still dramatic gaps in degree completion for first generation college students and students from low-income backgrounds. In particular, students enrolled in college who are both low-income and first-generation college are 36% less likely to obtain their bachelor’s degree than are students who are neither low-income nor first-generation college. Race/ethnic minority students tend to be overrepresented among low-income and first-generation students. Thus, sustained efforts to address race/ethnic and socioeconomic disparities in college enrollment and bachelor’s degree completion, such as the Dell Scholars Program (Page et al. 2016) and other powerful interventions that target key psychological barriers to college success (Aronson et al. 2002; Walton and Cohen 2007 and 2011), show promise for increasing the representation of students from race/ethnic minority and socioeconomically disadvantaged backgrounds in high-level STEMM and non-STEMM fields.

Within the middle-skill workforce, the pattern is starkly different. The same ethnic minority populations that are discussed as traditionally underrepresented in STEMM are actually overrepresented in STEMM middle-skill careers compared to non-STEMM middle-skill careers. So, within the middle-skill workforce individuals from these ethnic minority populations there is greater preference for working in STEMM than non-STEMM. Together, this suggests that the underrepresentation of some ethnic minority populations in STEMM careers is the result of differences in educational attainment, not discrepancies in preferences for STEMM careers.

When it comes to the underrepresentation of women in STEMM fields, the story is quite different. Men are consistently more likely than women to work in STEMM than non-STEMM careers across all skill-levels. Thus, gender differences are less likely to be attributed to educational attainment and more likely to derive from differences in preferences or other pressures that drive women away from STEMM careers. There are many factors that influence why some students are not attracted to certain STEMM careers. For example, perceptions of computer science among adolescent females are often negative with some seeing it as a boring subject devoid of interesting applications, computing careers as menial, that it is a boy’s domain, and that it is an individual or anti-social domain (e.g., Graham and Latulipe 2003, Varma and Lafever 2007, Wilson 2003). Given these perceptions, it is not surprising that the percentage of females working in computer fields actually declined from 34 to 27% between 1990 and 2011 (Landivar 2013). Compounding the problem, educators often ask learners to acquire knowledge with little context or with contexts that have little meaning to them (DeClue, 2009). Since female students bring contextual concerns to their learning (Fisher and Margolis 2002), this lack of relevance can be a strong deterrent from STEMM fields. Further, much of the literature on career choice points out a highly salient fact that is far from gender neutral: career decisions are made in early adulthood within the context of other life course events (e.g., having children, getting married) (Kerckhoff 1993; Clausen 1986). As Xie, Shauman, and Shauman (2003) argue, “gender differences in family expectations and the demands of familial roles may have a significant impact on the timing and sequencing of women’s science careers.” (p. 9).

Thus, increasing the representation of women in STEMM fields will require working to increase women’s preferences for STEMM fields changing the culture of STEMM fields to be more attractive to women, and structuring STEMM career entries and advancements recognizing contextual life events. However, to promote greater participation of individuals from traditionally underrepresented ethnic minority groups in STEMM, programs that support choices toward four-year college degree attainment or changes in the culture of higher education to be more inviting toward individuals from traditionally underrepresented ethnic minority groups are more likely to be successful.

This study considers an often-ignored proportion of the STEMM workforce, middle-skills workers. It does so by examining extant data. Analysis of extant data is inherently restricted to the data that were collected. For example, the data did not include measures specific of mathematics ability, spatial ability, or modern conceptions of scientific ability that integrate science knowledge with science practices. We did include many common predictors of the STEMM workforce. Those predictors were generally identified through study of the high-skill workforce. Relying on extant longitudinal data precludes causal claims of the influence of these variables on our outcome measures. Further, the data collection ended when respondents were 26 years old, an early time in their career trajectory, implying that they may later switch between STEMM and non-STEMM positions or from middle-skill to high-skill levels. A replication of these data using a more recent national dataset (e.g., Educational Longitudinal Study: 2002, ELS:02) may be warranted. The ELS:02 started in 10th grade rather than in 8th grade and the workforce placement of individuals in that dataset is likely to be highly influenced by the economic downturn in 2008, thus reducing the role of individual agency in career choice.

## Abbreviations

ELS:02: Educational Longitudinal Study of 2002
NELS:88: National Educational Longitudinal Study of 1988
O*NET: Occupational Information Network
PCAST: President’s Council of Advisors on Science and Technology
SCCT: Social Cultural Career Theory
STEM: Science, technology, engineering, and mathematics
STEMM: Science, technology, engineering, mathematics, and medicine
US: United States
